# Translational process

**DOI:** 10.1186/s12967-023-04507-7

**Published:** 2023-09-28

**Authors:** Marina Boccardi

**Affiliations:** 1 Deutsches Zentrum für Neurodegenerative Erkrankungen (DZNE), Rostock-Greifswald Standort, Rostock, Germany; 2 Department of Psychosomatic Medicine and Centre for Transdisciplinary Neurosciences, Rostock University of Medicine, Rostock, Germany

I am delighted to introduce a new section of the Journal of Translational Medicine: *Translational Process*. The section welcomes contributions aimed to streamline the transition of new knowledge along the translational continuum, and boost our ability to concretely innovate clinical practice.

Too often, clinical translational studies employ methods that are perfectly valid in basic research, but enable only a very slow translation along the continuum heading to clinics. Standard operating procedures are disregarded in the name of “freedom of research”. Requirements and constraints set by regulators, health technology assessment experts and policy makers seldom affect research priorities and study designs. Implementation is seen as a late, downstream and disconnected event entirely up to clinicians or other end-users. Despite increasing involvement of patients in research, frameworks and tools enabling effective interactions of the *multiple* stakeholders required to bring innovation are scantly available in the academic biomedical field. Interactions between academia and industry are difficult even at pre-competitive levels [1–6].

Unsurprisingly, the attrition rate of biomedical research is greater than 90% across different diseases [7]. 85% of the biomarker qualification procedures ever submitted to the EMA for any medical field failed, mostly due to gaps at very early development steps [8]. Clinical guidelines must be defined by expert consensus despite extensive literature, failing to demonstrate clinical validity [2, 9]. Besides delaying benefits to patients, such inconsistent proceeding results in high costs to society, investing in translational research that may be more efficient.

General frameworks for more efficient translation exist [10–12], but concrete projects converging needs and constraints from heterogeneous stakeholders are still sparse [13]. Specific definitions of the translational steps from bench to bed-side are available for many fields [14], but are not consistently followed, also due to lack of co-development with relevant stakeholders. Some regulators offer services and initiatives to increase interaction with researchers, but these are scantly known and used, or treat issues at too a high level to achieve concrete impact. The biomedical academic ecosystem may not dispose of the same clarity of objectives and system of incentives characterizing the technology field. This aspect makes *Translational Process* particularly complementary to the *Ecosystems* section in this Journal [15].

At the same time, ambitious projects and grant frameworks do aim to bring innovation, rightly leveraging interactions between academia and industry (e.g., the current European Innovative Health Initiative). However, consistent with pragmatic industrial practices and the lack of a specific discipline representing the field, much of the performed work is not published in scientific journals, hurdling retrieval to others needing similar methods. Efforts are thus duplicated, not leveraged upon, or inconsistent with each-other. Similarly, conflicts of interest go unchecked, lacking a framework seeking to capture those beyond direct involvement with pharma companies.

Do we dispose of well-defined translational methods? How can such methods be co-defined with relevant stakeholders, incorporating their needs, requirements and constraints at all development steps? How can new such methods be implemented among academic researchers? How can we dynamically assess the quality of our proceeding, monitor our action, and adjust it as needed, limiting waste and attrition? How can we capture and protect the translational process from the wider range of conflicts of interest scattered on its route?

This section dedicates a space to those who tackle such challenges overcoming the boundaries of individual disciplines. Through a high-standard peer-review process, the Journal of Translational Medicine offers a new opportunity to share and accelerate the development of urgently needed methods, tools and procedures. We warmly look forward to receiving your contributions and streamline the journey along the translational continuum.
